# Supplementary material for: Are carbon emissions trading and green financial instruments synergistic? -Comprehensive quantitative research based on content analysis
Source: PLoS One. 2024 Mar 7;19(3):e0298601. doi: 10.1371/journal.pone.0298601 (PMC10919601; doi:10.1371/journal.pone.0298601)
Supplement: S1 Data — (ZIP) [file pone.0298601.s001.zip › Data/地级市Z_F工作报告.zip!/║■▒▒/╗╞╩»╩╨/└┤╫╘╣1⁄2╓┌║┼í╛┬φ┐╦╩2╛▌═°í┐_2010.docx]

各位代表：

　　现在，我代表国务院向大会作政府工作报告，请予审议，并请全国政协各位委员提出意见。

　　一、2009年工作回顾

　　2009年是新世纪以来我国经济发展最为困难的一年。去年这个时候，国际金融危机还在扩散蔓延，世界经济深度衰退，我国经济受到严重冲击，出口大幅下降，不少企业经营困难，有的甚至停产倒闭，失业人员大量增加，农民工大批返乡，经济增速陡然下滑。在异常困难的情况下，全国各族人民在中国共产党的坚强领导下，坚定信心，迎难而上，顽强拼搏，从容应对国际金融危机冲击，在世界率先实现经济回升向好，改革开放和社会主义现代化建设取得新的重大成就。国内生产总值达到33.5万亿元，比上年增长8.7%；财政收入6.85万亿元，增长11.7%；粮食产量53082万吨，再创历史新高，实现连续6年增产；城镇新增就业1102万人；城镇居民人均可支配收入17175元，农村居民人均纯收入5153元，实际增长9.8%和8.5%。我国在全面建设小康社会道路上又迈出坚实的一步。实践再次证明，任何艰难险阻都挡不住中华民族伟大复兴的历史进程。

　　过去的一年，极不平凡，令人振奋。我们隆重庆祝新中国成立60周年。抚今追昔，伟大祖国的辉煌成就极大地激发了全国人民的自信心和自豪感，极大地增强了中华民族的向心力和凝聚力，极大地提升了我国的国际地位和影响力，必将激励我们在中国特色社会主义道路上继续奋勇前进。

　　一年来，我们主要做了以下工作：

　　（一）加强和改善宏观调控，促进经济平稳较快发展。我们实行积极的财政政策和适度宽松的货币政策，全面实施并不断完善应对国际金融危机的一揽子计划。大规模增加财政支出和实行结构性减税，保持货币信贷快速增长，提高货币政策的可持续性，扩大直接融资规模，满足经济社会发展的资金需求，有效扩大了内需，很快扭转了经济增速下滑趋势。

　　着力扩大居民消费。我们鼓励消费的政策领域之宽、力度之大、受惠面之广前所未有。中央财政投入资金450亿元，补贴家电汽车摩托车下乡、汽车家电以旧换新和农机具购置。减半征收小排量汽车购置税，减免住房交易相关税收，支持自住性住房消费。全年汽车销售1364万辆，增长46.2%；商品房销售9.37亿平方米，增长42.1%；社会消费品零售总额实际增长16.9%，消费对经济增长的拉动作用明显增强。

　　促进投资快速增长。我们发挥政府投资“四两拨千斤”的作用，引导带动社会投资。实施两年新增4万亿元的投资计划。2009年中央政府公共投资9243亿元，比上年预算增加5038亿元，其中，保障性住房、农村民生工程、社会事业投资占44%，自主创新、结构调整、节能减排和生态建设占16%，重大基础设施建设占23%，灾后恢复重建占14%。全社会固定资产投资增长30.1%，投资结构进一步优化。投资快速增长有效弥补了外需下降的缺口，加强了薄弱环节，为经济社会长远发展奠定了坚实的基础。

　　我们加快推进汶川地震灾后恢复重建，重灾区已完成投资6545亿元，占规划总投资的65.5%。国家的大力支持，全国人民的无私援助，灾区群众的艰苦奋斗，使遭受重大创伤的灾区呈现出崭新面貌。一座座新城拔地而起，一个个村庄焕发出蓬勃生机。这充分体现了中华民族的无疆大爱，有力彰显了社会主义制度的无比优越。

　　（二）大力调整经济结构，夯实长远发展基础。我们把保增长与调结构紧密结合起来，加快解决制约经济发展的结构性矛盾。

　　“三农”工作进一步加强。中央财政用于“三农”的支出7253亿元，增长21.8%。大幅度提高粮食最低收购价。启动实施全国新增千亿斤粮食生产能力建设规划。继续改善农村生产生活条件，农村饮水安全工程使6069万农民受益，新增510万沼气用户，新建和改造农村公路38万公里、农村电网线路26.6万公里，又有80万户农村危房得到改造，9.2万户游牧民实现了定居。我们加大扶贫力度，贫困地区的生产生活条件得到明显改善。

　　产业结构调整力度加大。制定并实施十大重点产业调整振兴规划。鼓励企业加快技术改造，安排200亿元技改专项资金支持4441个技改项目。重点行业兼并重组取得新进展。下大力气抑制部分行业产能过剩和重复建设，关停小火电机组2617万千瓦，淘汰落后的炼钢产能1691万吨、炼铁产能2113万吨、水泥产能7416万吨、焦炭产能1809万吨。加快实施国家科技重大专项，中央财政用于科技的支出1512亿元，增长30%。积极支持自主创新产品推广应用，清洁能源、第三代移动通信等一批新兴产业快速发展。大力加强基础设施建设，新建铁路投入运营5557公里，高速公路新建通车4719公里，城市轨道交通建设加快，新建、改扩建民用机场35个；新增发电装机8970万千瓦，西气东输二线西段工程实现供气，南水北调工程建设加快推进，6183座病险水库除险加固工程开工建设。

　　节能减排和环境保护扎实推进。安排预算内资金，支持重点节能工程、循环经济等项目2983个；实施节能产品惠民工程，推广节能空调500多万台、高效照明灯具1.5亿只。继续推进林业重点生态工程建设，完成造林588万公顷，森林覆盖率达到20.36%。综合治理水土流失面积4.8万平方公里。加强“三河三湖”等重点流域水污染防治和工业废水废气废渣治理。“十一五”前四年累计单位国内生产总值能耗下降14.38%，化学需氧量、二氧化硫排放量分别下降9.66%和13.14%。积极开展应对气候变化工作，明确提出2020年我国控制温室气体排放行动目标和政策措施。气象预报预警和地震监测工作得到加强，灾害防御能力不断提升。

　　区域协调发展迈出新步伐。深入实施区域发展总体战略，制定若干区域发展重大规划和政策。中西部和东北地区加快开放开发，积极承接产业转移，发展基础不断夯实；东部地区加快结构调整和自主创新，经济发展活力增强。区域发展呈现布局改善、结构优化、协调性提高的良好态势。

　　（三）坚持深化改革开放，不断完善有利于科学发展的体制机制。我们把深化改革开放作为应对国际金融危机的强大动力，努力消除体制障碍，不断提高对外开放水平。

　　重点领域和关键环节改革加快推进。增值税转型全面实施。成品油价格和税费改革顺利推进，新的成品油价格形成机制规范运行。国家开发银行商业化转型和农业银行股份制改革扎实推进，跨境贸易人民币结算试点启动实施。创业板正式推出，为自主创新及其他成长型创业企业开辟了新的融资渠道。地方政府机构改革有序开展，事业单位分类改革试点稳步进行。集体林权制度改革全面推开，1亿公顷林地确权到户，占全国集体林地面积的60%，这是继土地家庭承包之后我国农村经营制度的又一重大变革。

　　开放型经济水平不断提高。出台一系列稳定外需的政策措施，采取符合国际惯例的方式支持出口企业，完成短期出口信用保险承保900亿美元，安排421亿美元大型成套设备出口融资保险。鼓励增加进口。去年下半年开始进出口降幅明显收窄，国际市场份额得到巩固，全年进出口总额2.2万亿美元。扭转利用外资下降局面，全年实际利用外商直接投资900亿美元。企业“走出去”逆势上扬，非金融类对外直接投资和对外工程承包营业额分别达433亿美元和777亿美元。积极参与国际宏观经济政策对话协调和经贸金融合作，在共同应对国际金融危机中发挥了建设性作用。

　　（四）着力改善民生，加快发展社会事业。在应对国际金融危机的困难情况下，我们更加注重保障和改善民生，切实解决人民群众最关心、最直接、最现实的利益问题。

　　实施更加积极的就业政策。强化政府促进就业的责任。中央财政安排就业专项资金426亿元，比上年增长59%。实施困难企业缓缴社会保险费或降低部分费率、再就业税收减免及提供相关补贴等政策，鼓励企业稳定和增加就业。开展系列就业服务活动，多渠道开辟公益性就业岗位，促进高校毕业生到基层就业、应征入伍和到企事业单位就业见习。全年组织2100万城乡劳动者参加职业培训。这些措施促进了就业的基本稳定。

　　加快完善社会保障体系。普遍建立养老保险省级统筹制度，出台包括农民工在内的城镇企业职工养老保险关系转移接续办法。在320个县开展新型农村社会养老保险试点，推动我国社会保障制度建设迈出历史性步伐。中央财政安排社会保障资金2906亿元，比上年增长16.6%。企业退休人员基本养老金连续5年增加，去年又人均提高10%。农村五保户供养水平、优抚对象抚恤补助标准、城乡低保对象保障水平都有新的提高。中央财政安排保障性安居工程补助资金551亿元，比上年增长2倍。新建、改扩建各类保障性住房200万套，棚户区改造解决住房130万套。全国社会保障基金积累6927亿元，比上年增长44.2%。社会保障体系得到加强。

　　进一步促进教育公平。大幅度增加全国教育支出，其中中央财政支出1981亿元，比上年增长23.6%。全面落实城乡义务教育政策，中央下达农村义务教育经费666亿元，提前一年实现农村中小学生人均公用经费500元和300元的目标。实行义务教育阶段教师绩效工资制度。中等职业学校农村家庭经济困难学生和涉农专业学生免学费政策开始实施。国家助学制度不断完善，资助学生2871万人，基本保障了困难家庭的孩子不因贫困而失学。

　　稳步推进医药卫生事业改革发展。组织实施医药卫生体制改革。中央财政医疗卫生支出1277亿元，比上年增长49.5%。城镇职工和城镇居民基本医疗保险参保4.01亿人，新型农村合作医疗制度覆盖8.3亿人。中央财政安排429亿元，解决关闭破产国有企业退休人员医疗保险问题。基本药物制度在30%的基层医疗卫生机构实施。中央财政支持建设了一批县级医院、乡镇中心卫生院和社区卫生服务中心。启动实施扩大乙肝疫苗接种等重大公共卫生服务专项。加强食品、药品安全专项整治。面对突如其来的甲型H1N1流感疫情，我们依法科学有序地开展防控工作，有效保障了人民群众生命安全，维护了社会正常秩序。

　　在国际金融危机严重冲击、世界经济负增长的背景下，我国取得这样的成绩极为不易。这是以胡锦涛同志为总书记的党中央统揽全局、正确领导的结果，是全党全军全国各族人民和衷共济、团结奋斗的结果。在这里，我代表国务院，向全国各族人民，各民主党派、各人民团体和各界人士，表示诚挚的感谢！向香港特别行政区同胞、澳门特别行政区同胞、台湾同胞和海外侨胞，表示诚挚的感谢！向关心和支持中国现代化建设的各国政府、国际组织和各国朋友，表示诚挚的感谢！

　　一年来，我们认真贯彻落实科学发展观，积极应对国际金融危机，全面做好政府工作，有以下几点体会：必须坚持运用市场机制和宏观调控两种手段，在坚持市场经济改革方向、发挥市场配置资源基础性作用、激发市场活力的同时，充分发挥我国社会主义制度决策高效、组织有力、集中力量办大事的优势。必须坚持处理好短期和长期两方面关系，注重远近结合、标本兼治，既克服短期困难、解决突出矛盾，又加强重点领域和薄弱环节、为长远发展奠定基础。必须坚持统筹国内国际两个大局，把扩大内需作为长期战略方针，坚定不移地实行互利共赢的开放战略，加快形成内需外需协调拉动经济增长的格局。必须坚持发展经济与改善民生、维护社会公平正义的内在统一，围绕改善民生谋发展，把改善民生作为经济发展的出发点、落脚点和持久动力，着眼维护公平正义，让全体人民共享改革发展成果，促进社会和谐稳定。必须坚持发挥中央和地方两个积极性，既强调统一思想、顾全大局，又鼓励因地制宜、探索创新，形成共克时艰的强大合力。这些经验对我们坚持中国特色社会主义道路，提高驾驭社会主义市场经济能力，推进现代化进程，具有重要而深远的意义。

　　二、2010年主要任务

　　今年是继续应对国际金融危机、保持经济平稳较快发展、加快转变经济发展方式的关键一年，是全面实现“十一五”规划目标、为“十二五”发展打好基础的重要一年。

　　今年发展环境虽然有可能好于去年，但是面临的形势极为复杂。各种积极变化和不利影响此长彼消，短期问题和长期矛盾相互交织，国内因素和国际因素相互影响，经济社会发展中“两难”问题增多。从国际看，世界经济有望恢复性增长，国际金融市场渐趋稳定，经济全球化深入发展的大趋势没有改变，世界经济格局大变革、大调整孕育着新的发展机遇。同时，世界经济复苏的基础仍然脆弱，金融领域风险没有完全消除，各国刺激政策退出抉择艰难，国际大宗商品价格和主要货币汇率可能加剧波动，贸易保护主义明显抬头，加上气候变化、粮食安全、能源资源等全球性问题错综复杂，外部环境不稳定、不确定因素依然很多。从国内看，我国仍处在重要战略机遇期。经济回升向好的基础进一步巩固，市场信心增强，扩大内需和改善民生的政策效应继续显现，企业适应市场变化的能力和竞争力不断提高。但是，经济社会发展中仍然存在一些突出矛盾和问题。经济增长内生动力不足，自主创新能力不强，部分行业产能过剩矛盾突出，结构调整难度加大；就业压力总体上持续增加和结构性用工短缺的矛盾并存；农业稳定发展和农民持续增收的基础不稳固；财政金融领域潜在风险增加；医疗、教育、住房、收入分配、社会管理等方面的突出问题亟待解决。我们必须全面、正确判断形势，决不能把经济回升向好的趋势等同于经济运行根本好转。要增强忧患意识，充分利用有利条件和积极因素，努力化解矛盾，更加周密地做好应对各种风险和挑战的准备，牢牢把握工作的主动权。

　　做好今年的政府工作，要认真贯彻党的十七大和十七届三中、四中全会精神，以邓小平理论和“三个代表”重要思想为指导，深入贯彻落实科学发展观，着力搞好宏观调控和保持经济平稳较快发展，着力加快经济发展方式转变和经济结构调整，着力推进改革开放和自主创新，着力改善民生和促进社会和谐稳定，全面推进社会主义经济建设、政治建设、文化建设、社会建设以及生态文明建设，加快全面建设小康社会进程，努力实现经济社会又好又快发展。

　　今年经济社会发展的主要预期目标是：国内生产总值增长8%左右；城镇新增就业900万人以上，城镇登记失业率控制在4.6%以内；居民消费价格涨幅3%左右；国际收支状况改善。这里要着重说明，提出国内生产总值增长8%左右，主要是强调好字当头，引导各方面把工作重点放到转变经济发展方式、调整经济结构上来。提出居民消费价格涨幅3%左右，综合考虑了去年价格变动的翘尾因素、国际大宗商品价格的传导效应、国内货币信贷增长的滞后影响以及居民的承受能力，并为资源环境税费和资源性产品价格改革留有一定空间。

　　今年要重点抓好八个方面工作：

　　（一）提高宏观调控水平，保持经济平稳较快发展

　　要继续实施积极的财政政策和适度宽松的货币政策，保持政策的连续性和稳定性，根据新形势新情况不断提高政策的针对性和灵活性，把握好政策实施的力度、节奏和重点。处理好保持经济平稳较快发展、调整经济结构和管理通胀预期的关系。既要保持足够的政策力度、巩固经济回升向好的势头，又要加快经济结构调整、推动经济发展方式转变取得实质性进展，还要管理好通胀预期、稳定物价总水平。

　　继续实施积极的财政政策。一是保持适度的财政赤字和国债规模。今年拟安排财政赤字10500亿元，其中中央财政赤字8500亿元，继续代发地方债2000亿元并纳入地方财政预算。这样的安排主要是考虑到今年财政收支矛盾仍然十分突出。从财政收入看，上年一次性特殊增收措施没有或减少了，还要继续实施结构性减税政策，财政收入增长不会太快；从财政支出看，继续实施应对国际金融危机的一揽子计划，完成在建项目、加强薄弱环节、推进改革、改善民生、维护稳定等都需要增加投入。二是继续实施结构性减税政策，促进扩大内需和经济结构调整。三是优化财政支出结构，有保有压，把钱花在刀刃上。继续向“三农”、民生、社会事业等领域倾斜，支持节能环保、自主创新和欠发达地区的建设。严格控制一般性支出，大力压缩公用经费。四是切实加强政府性债务管理，增强内外部约束力，有效防范和化解潜在财政风险。同时，要依法加强税收征管和非税收入管理，严厉打击偷骗税行为，做到应收尽收。

　　继续实施适度宽松的货币政策。一是保持货币信贷合理充裕。今年广义货币M2增长目标为17%左右，新增人民币贷款7.5万亿元左右。这两个指标虽然都低于去年实际执行结果，但仍然是适度宽松的政策目标，能够满足经济社会发展的合理资金需求。同时也有利于管理好通胀预期、提高金融支持经济发展的可持续性。二是优化信贷结构。落实有保有控的信贷政策，加强对重点领域和薄弱环节的支持，有效缓解农户和小企业融资难问题，严格控制对“两高”行业和产能过剩行业的贷款。强化贷后管理，确保信贷资金支持实体经济。三是积极扩大直接融资。完善多层次资本市场体系，扩大股权和债券融资规模，更好地满足多样化投融资需求。四是加强风险管理，提高金融监管有效性。探索建立宏观审慎管理制度，强化对跨境资本流动的有效监控，防范各类金融风险。继续完善人民币汇率形成机制，保持人民币汇率在合理、均衡水平上的基本稳定。

　　积极扩大居民消费需求。继续提高农民收入、企业退休人员基本养老金、部分优抚对象待遇和城乡居民最低生活保障水平，增强居民特别是中低收入者消费能力。巩固扩大传统消费，积极培育信息、旅游、文化、健身、培训、养老、家庭服务等消费热点，促进消费结构优化升级。扩大消费信贷。加强商贸流通体系等基础设施建设，积极发展电子商务。整顿和规范市场秩序，努力营造便利、安全、放心的消费环境。继续实施和完善鼓励消费的各项政策措施。大幅提高家电下乡产品最高限价，增加品种和型号，扩大补贴范围，完善补贴标准和办法，加强对中标企业的管理和考核，提高产品质量和服务水平；完善家电、汽车以旧换新和汽车、摩托车下乡政策，小排量汽车购置税按7.5%征收。我们一定要落实好这些政策措施，把好事办好，真正让广大群众得到实惠。

　　着力优化投资结构。各级政府投资都要集中力量保重点，严格控制新开工项目，资金安排主要用于项目续建和收尾，切实防止出现“半拉子”工程。扎实推进地震灾区恢复重建，保质保量完成任务。鼓励扩大民间投资，完善和落实促进民间投资的相关政策。加强和改进投资管理，严格执行用地、节能、环保、安全等市场准入标准和产业政策，切实防止重复建设。对有财政资金投入的建设项目，要加强全程监督，坚决避免以扩大内需为名，搞劳民伤财的形象工程和政绩工程。坚持科学民主决策，确保公共投资真正用于推进经济社会发展和改善人民生活，经得起实践和历史的检验。

　　（二）加快转变经济发展方式，调整优化经济结构

　　转变经济发展方式刻不容缓。要大力推动经济进入创新驱动、内生增长的发展轨道。

　　继续推进重点产业调整振兴。一是加大技术改造力度。用好技改专项资金，引导企业开发新产品和节能降耗。二是促进企业兼并重组。打破行业垄断和地区封锁，推动优势企业兼并困难企业，加快淘汰落后产能。三是全面提升产品质量。引导企业以品牌、标准、服务和效益为重点，健全质量管理体系，强化社会责任。切实加强市场监管和诚信体系建设，努力把我国产品质量提高到新水平。

　　大力培育战略性新兴产业。国际金融危机正在催生新的科技革命和产业革命。发展战略性新兴产业，抢占经济科技制高点，决定国家的未来，必须抓住机遇，明确重点，有所作为。要大力发展新能源、新材料、节能环保、生物医药、信息网络和高端制造产业。积极推进新能源汽车、“三网”融合取得实质性进展，加快物联网的研发应用。加大对战略性新兴产业的投入和政策支持。

　　进一步促进中小企业发展。一是建立和完善中小企业服务体系。抓紧修订中小企业划分标准，加快中小企业公共服务平台、信息服务网络和小企业创业基地建设，进一步减少、简化行政审批，坚决清理和取消不合理收费。二是继续落实财政对中小企业支持政策。中央财政扶持中小企业发展专项资金安排106亿元。对部分小型微利企业实行所得税优惠政策。中央财政预算内技术改造专项投资要覆盖中小企业，地方政府也要加大投入。三是加强对中小企业的金融支持。完善小企业信贷考核体系。鼓励建立小企业贷款风险补偿基金。中小企业贷款税前全额拨备损失准备金。发展多层次中小企业信用担保体系，落实好对符合条件的中小企业信用担保机构免征营业税、准备金提取和代偿损失在税前扣除的政策。拓宽中小企业融资渠道，切实解决中小企业特别是小企业融资难问题。

　　加快发展服务业。进一步提高服务业发展水平和在国民经济中的比重。大力发展金融、物流、信息、研发、工业设计、商务、节能环保服务等面向生产的服务业，促进服务业与现代制造业有机融合。大力发展市政公用事业、房地产和物业服务、社区服务等面向民生的服务业，加快发展旅游业，积极拓展新型服务领域。农村服务业基础薄弱、发展潜力大，要加快构建和完善以生产销售、科技信息和金融服务为主体的农村生产生活服务体系。加快建立公开平等规范的服务业准入制度，鼓励社会资本进入。进一步完善促进服务业发展的政策，逐步实现国家鼓励类服务业用电、用水、用气、用热与工业基本同价。

　　打好节能减排攻坚战和持久战。一要以工业、交通、建筑为重点，大力推进节能，提高能源效率。扎实推进十大重点节能工程、千家企业节能行动和节能产品惠民工程，形成全社会节能的良好风尚。今年要新增8000万吨标准煤的节能能力。所有燃煤机组都要加快建设并运行烟气脱硫设施。二要加强环境保护。积极推进重点流域区域环境治理及城镇污水垃圾处理、农业面源污染治理、重金属污染综合整治等工作。新增城镇污水日处理能力1500万立方米、垃圾日处理能力6万吨。三要积极发展循环经济和节能环保产业。支持循环经济技术研发、示范推广和能力建设。抓好节能、节水、节地、节材工作。推进矿产资源综合利用、工业废物回收利用、余热余压发电和生活垃圾资源化利用。合理开发利用和保护海洋资源。四要积极应对气候变化。加强适应和减缓气候变化的能力建设。大力开发低碳技术，推广高效节能技术，积极发展新能源和可再生能源，加强智能电网建设。加快国土绿化进程，增加森林碳汇，新增造林面积不低于592万公顷。要努力建设以低碳排放为特征的产业体系和消费模式，积极参与应对气候变化国际合作，推动全球应对气候变化取得新进展。

　　推进区域经济协调发展。继续深入推进西部大开发，全面振兴东北地区等老工业基地，大力促进中部地区崛起，积极支持东部地区率先发展。认真落实促进区域经济社会发展的各项规划和政策。加快推进主体功能区建设。加强对革命老区、民族地区、边疆地区和贫困地区的支持。重点抓好西藏和四省藏区、新疆经济社会发展政策的制定和实施工作。实施区域发展总体战略，重在发挥各地比较优势，有针对性地解决各地发展中的突出矛盾和问题；重在扭转区域经济社会发展差距扩大的趋势，增强发展的协调性；重在加快完善公共财政体系，促进基本公共服务均等化。

　　（三）加大统筹城乡发展力度，强化农业农村发展基础

　　在连续6年增产增收之后，更要毫不松懈地抓好“三农”工作。要按照统筹城乡发展的要求，坚持把解决好“三农”问题作为全部工作的重中之重，进一步强化强农惠农政策，协调推进工业化、城镇化和农业农村现代化，巩固和发展农业农村好形势。

　　促进农业稳定发展和农民持续增收。稳定粮食生产，扩大油料种植面积，增加重要紧缺农产品供应，大规模开展粮棉油糖高产创建，大规模开展园艺产品生产和畜牧水产养殖标准化创建，保障“米袋子”、“菜篮子”安全。继续实施对种粮农民直接补贴，增加农资综合补贴、良种补贴、农机具购置补贴，中央财政拟安排补贴资金1335亿元，比上年增加60.4亿元。进一步提高粮食最低收购价，早籼稻、中晚籼稻、粳稻每50公斤分别提高3元、5元和10元，小麦每50公斤提高3元，继续实施重要农产品临时收储政策，让农民得到更多实惠。加强对产粮大县、养猪大县、养牛大县的财政扶持。大力发展农产品加工业，推进农业产业化经营，支持批发市场和农贸市场升级改造，推动生产与市场对接。促进农民就业创业，多渠道增加农民收入。进一步加大扶贫开发力度。我们要坚持不懈地消除贫困落后，让农民群众早日过上富裕安康的生活。

　　加强农业基础设施建设。坚持财政支出优先支持农业农村发展，预算内固定资产投资优先投向农业基础设施和农村民生工程，土地出让收益优先用于农业土地开发和农村基础设施建设。中央财政拟安排“三农”投入8183亿元，比上年增加930亿元，地方各级财政也要增加投入。以主产区为重点，全面实施全国新增千亿斤粮食生产能力建设规划。以农田水利为重点，加强农业基础设施建设，加快大中型灌区的配套改造，扩大节水灌溉面积，建设高标准农田，完成大中型和重点小型病险水库除险加固任务。以良种培育为重点，加快农业科技创新和推广，实施好转基因生物新品种培育科技重大专项。积极推进现代农业示范区建设。加快建设乡镇和区域性农技推广、动植物疫病防控、农产品质量监管等公共服务机构。

　　深化农村改革。毫不动摇地坚持农村基本经营制度，加快完善有关法律法规和政策，现有土地承包关系要保持稳定并长久不变。加强土地承包经营权流转的管理和服务，在依法自愿有偿流转基础上发展多种形式规模经营。继续推进农村综合改革。完善集体林权制度改革配套政策。启动国有林场改革。继续推进草原基本经营制度改革。发展农民专业合作社，提高农业组织化程度。加快培育小型农村金融机构，积极推广农村小额信用贷款，切实改善农村金融服务。深入推进乡镇机构改革。

　　统筹推进城镇化和新农村建设。坚持走中国特色城镇化道路，促进大中小城市和小城镇协调发展，着力提高城镇综合承载能力，发挥城市对农村的辐射带动作用，促进城镇化和新农村建设良性互动。壮大县域经济，大力加强县城和中心镇基础设施和环境建设，引导非农产业和农村人口有序向小城镇集聚，鼓励返乡农民工就地创业。城乡建设都要坚持最严格的耕地保护制度和最严格的节约用地制度，切实保护农民合法权益。推进户籍制度改革，放宽中小城市和小城镇落户条件。有计划有步骤地解决好农民工在城镇的就业和生活问题，逐步实现农民工在劳动报酬、子女就学、公共卫生、住房租购以及社会保障方面与城镇居民享有同等待遇。进一步增加农村生产生活设施建设投入，启动新一轮农村电网改造，扩大农村沼气建设规模，今年再解决6000万农村人口的安全饮水问题，实施农村清洁工程，改善农村生产生活条件。我们要让符合条件的农业转移人口逐步变为城镇居民，也要让农民有一个幸福生活的美好家园。

　　（四）全面实施科教兴国战略和人才强国战略

　　教育、科技和人才，是国家强盛、民族振兴的基石，也是综合国力的核心。

　　优先发展教育事业。强国必先强教。只有一流的教育，才能培养一流人才，建设一流国家。要抓紧启动实施国家中长期教育改革和发展规划纲要。着重抓好五个方面：一是推进教育改革。要解放思想，大胆突破，勇于创新，鼓励试验，对办学体制、教学内容、教育方法、评价制度等进行系统改革。坚持育人为本，大力推进素质教育。探索适应不同类型教育和人才成长的学校管理体制和办学模式，提高办学和人才培养水平。鼓励社会力量兴办教育，满足群众多样化的教育需求。二是促进义务教育均衡发展。在合理布局的基础上，加快推进中西部地区初中校舍改造和全国中小学校舍安全工程，尽快使所有学校的校舍、设备和师资达到规定标准。为农村中小学班级配备多媒体远程教学设备，让广大农村和偏远地区的孩子共享优质教育资源。加强学前教育和特殊教育学校建设。加大对少数民族和民族地区教育的支持。三是继续加强职业教育。以就业为目标，整合教育资源，改进教学方式，着力培养学生的就业创业能力。四是推进高等学校管理体制和招生制度改革。进一步落实高等学校办学自主权，鼓励高等学校适应就业和经济社会发展需要，调整专业和课程设置，推动高等学校人才培养、科技创新和学术发展紧密结合，激励教师专注于教育，努力建设有特色、高水平大学。创建若干一流大学，培养杰出人才。中央财政要加大对中西部高等教育发展的支持。五是加强教师队伍建设。从多方面采取措施，吸引优秀人才投身教育事业，鼓励他们终身从教。重点加强农村义务教育学校教师和校长培训，鼓励优秀教师到农村贫困地区从教。加强师德教育，增强教师的责任感和使命感。教育寄托着亿万家庭对美好生活的期盼，关系着民族素质和国家未来。教育不普及不提高，国家不可能强盛。这个道理我们要永远铭记。

　　（下转第７版）

　　大力发展科学技术。要认真贯彻自主创新的方针，全面推进创新型国家建设。加快实施科技重大专项，着力突破带动技术革命、促进产业振兴的关键科技问题，突破提高健康水平、保障改善民生的重大公益性科技问题，突破增强国际竞争力、维护国家安全的战略高技术问题。前瞻部署生物、纳米、量子调控、信息网络、气候变化、空天海洋等领域基础研究和前沿技术研究。深化科技体制改革，着力解决科技与经济脱节的问题，推动以企业为主体、市场为导向、产学研相结合的技术创新体系建设，促进科技资源优化配置、开放共享和高效利用。要大力实施知识产权战略，加强知识产权创造、应用和保护。进一步激发广大科技工作者和全社会的创新活力。

　　加快人才资源开发。人才是第一资源。要统筹推进各类人才队伍建设，突出培养创新型科技人才、经济社会发展重点领域专门人才和高技能人才，积极引进海外高层次人才。建立健全政府、社会、用人单位和个人等多元化的人才培养投入机制，充分发挥市场配置人才资源的基础性作用，努力营造人才辈出、人尽其才的制度环境，建设人力资源强国。

　　（五）大力加强文化建设

　　国家发展、民族振兴，不仅需要强大的经济力量，更需要强大的文化力量。文化是一个民族的精神和灵魂，是一个民族真正有力量的决定性因素，可以深刻影响一个国家发展的进程，改变一个民族的命运。没有先进文化的发展，没有全民族文明素质的提高，就不可能真正实现现代化。解放思想、改革开放的时代精神，已经成为推动社会前进的先进文化力量，使中华民族充满生机与活力。

　　一年来，我们大力发展公益性文化事业，加快推进文化体制改革，加强公共文化服务体系建设，促进文化产业快速成长，繁荣文化市场，有效扩大了内需。新的一年，我们要更加重视和大力加强文化建设。继承和弘扬中华民族优秀传统文化，吸收和借鉴世界各国文明成果，建设中华民族共有精神家园。政府要更好地履行发展公益性文化事业的责任，保障人民群众的基本需求和权益。文化基础设施建设和公共文化资源配置要向基层、特别是农村和中西部地区倾斜，推进美术馆、图书馆、文化馆、博物馆免费开放，丰富人民群众的精神文化生活。要继续推进文化体制改革，扶持公益性文化事业，发展文化产业，鼓励文化创新，培育骨干文化企业，生产更多健康向上的文化产品，满足人民群众多样化的文化需求。促进哲学社会科学、广播影视、新闻出版、档案事业发展，繁荣文学艺术创作，加强文物和非物质文化遗产保护。积极开展对外文化交流，增强中华文化国际影响力。大力发展公共体育事业，广泛开展全民健身运动，提高人民的身体素质。中华民族不仅能够创造经济奇迹，也一定能够创造新的文化辉煌。

　　（六）着力保障和改善民生，促进社会和谐进步

　　改善民生是经济发展的根本目的。只有着力保障和改善民生，经济发展才有持久的动力，社会进步才有牢固的基础，国家才能长治久安。

　　千方百计扩大就业。这是保障和改善民生的头等大事。今年就业形势依然严峻，工作上不能有丝毫松懈。要继续实施积极的就业政策。中央财政拟投入433亿元用于促进就业。重点做好高校毕业生、农民工、就业困难人员就业和退伍转业军人就业安置工作。2009年到期的“五缓四减三补贴”就业扶持政策延长一年。加强政策支持和就业指导，鼓励高校毕业生到城乡基层、中西部地区和中小企业就业；拓宽就业、择业、创业渠道，鼓励自主创业、自谋职业等多种形式的灵活就业，以创业带动就业。建立健全公共投资带动就业的机制。继续加强职业技能培训，重点提高农民工和城乡新增劳动力的就业能力。完善就业服务体系，健全劳动力输出输入地区协调协作机制，引导劳动力特别是农民工有序流动。加快建立统一规范的人力资源市场。维护劳动者合法权益，构建和谐的劳动关系。我们要通过持之以恒的努力，创造更多的就业机会，让广大劳动者各尽所能、各得其所。

　　加快完善覆盖城乡居民的社会保障体系。扎实推进新型农村社会养老保险试点，试点范围扩大到23%的县。加快解决未参保集体企业退休人员基本养老保障等遗留问题。将全国130万“老工伤”人员全部纳入工伤保险范围。积极推进农民工参加社会保险。加强城乡低保工作，逐步提高保障水平，切实做到动态管理、应保尽保。加强残疾人社会保障和服务体系建设，进一步落实好扶残助残的各项政策，为他们平等参与社会生活创造更好的环境。企业退休人员基本养老金今年再提高10%。各级政府要进一步增加社会保障投入，中央财政拟安排3185亿元。要多渠道增加全国社会保障基金，加强监管，实现保值增值。鼓励和支持慈善事业发展。我们要加快构建更加完善的社会保障安全网，使人民生活有基本保障、无后顾之忧。

　　改革收入分配制度。合理的收入分配制度是社会公平正义的重要体现。我们不仅要通过发展经济，把社会财富这个“蛋糕”做大，也要通过合理的收入分配制度把“蛋糕”分好。要坚持和完善按劳分配为主体、多种分配方式并存的分配制度，兼顾效率与公平，走共同富裕的道路。一要抓紧制定调整国民收入分配格局的政策措施，逐步提高居民收入在国民收入分配中的比重，提高劳动报酬在初次分配中的比重。加大财政、税收在收入初次分配和再分配中的调节作用。创造条件让更多群众拥有财产性收入。二要深化垄断行业收入分配制度改革。完善对垄断行业工资总额和工资水平的双重调控政策。严格规范国有企业、金融机构经营管理人员特别是高管的收入，完善监管办法。三要进一步规范收入分配秩序。保护合法收入，调节过高收入，取缔非法收入，逐步形成公开透明、公正合理的收入分配秩序，坚决扭转收入差距扩大的趋势。

　　促进房地产市场平稳健康发展。要坚决遏制部分城市房价过快上涨势头，满足人民群众的基本住房需求。一是继续大规模实施保障性安居工程。中央财政拟安排保障性住房专项补助资金632亿元，比上年增加81亿元。建设保障性住房300万套，各类棚户区改造住房280万套。扩大农村危房改造试点范围。各级政府要切实负起责任，严格执行年度建设计划，确保土地、资金和优惠政策落实到位。二是继续支持居民自住性住房消费。增加中低价位、中小套型普通商品房用地供应，加快普通商品房项目审批和建设进度。规范发展二手房市场，倡导住房租赁消费。盘活住房租赁市场。三是抑制投机性购房。加大差别化信贷、税收政策执行力度。完善商品房预售制度。四是大力整顿和规范房地产市场秩序。完善土地收入管理使用办法，抑制土地价格过快上涨。加大对圈地不建、捂盘惜售、哄抬房价等违法违规行为的查处力度。

　　加快推进医药卫生事业改革发展。积极稳妥推进医药卫生体制改革，全面落实五项重点工作。继续扩大基本医疗保障覆盖面。今年要把城镇居民基本医保和新农合的财政补助标准提高到120元，比上年增长50%，并适当提高个人缴费标准。开展农村儿童白血病、先天性心脏病医疗保障试点，尽力为这些不幸的儿童和家庭提供更多帮助。在60%政府举办的基层医疗卫生机构实施基本药物制度，其他医疗机构也要优先选用基本药物。推进基本药物集中采购和统一配送。基本完成城乡基层医疗卫生机构建设规划，大规模开展适宜人才培养和培训。进一步完善支持村卫生室建设和乡村医生发展的政策措施。完善基层医疗卫生机构补偿机制，落实岗位绩效工资。开展社区首诊试点，推动形成基层医疗卫生机构和医院功能区分合理、协作配合、互相转诊的服务体系。切实加强甲型H1N1流感等重大传染病防控和慢性病、职业病、地方病防治，提高突发公共卫生事件应急处置能力。开展公立医院改革试点，坚持基本医疗的公益性方向，创新体制机制，充分调动医务人员积极性，提高服务质量，控制医疗费用，改善医患关系。大力支持社会资本兴办医疗卫生机构，在服务准入、医保定点等方面一视同仁。扶持和促进中医药、民族医药事业发展。医药卫生事业改革发展关系人民身体健康和家庭幸福，我们要克服一切困难，把这个世界性难题解决好。

　　做好人口和计划生育工作。继续稳定低生育水平。做好流动人口计划生育服务工作。落实好农村妇女妇科疾病定期检查和住院分娩补助政策。加强出生缺陷干预，开展免费孕前优生健康检查试点，做好孕产妇和婴幼儿保健工作。继续实施农村部分计划生育家庭奖励扶助制度和西部地区少生快富工程。切实保护好妇女和未成年人权益。加强应对人口老龄化战略研究，加快建立健全养老社会服务体系，让老年人安享晚年生活。

　　（七）坚定不移推进改革，进一步扩大开放

　　今年要继续深化重点领域和关键环节改革，努力实现新的突破。

　　我们要继续推进国有经济布局和结构战略性调整。加快大型国有企业特别是中央企业母公司的公司制改革，实现产权多元化，完善法人治理结构。加快推进垄断性行业改革，推进公用事业改革，切实放宽市场准入，积极引入竞争机制。着力营造多种所有制经济公平竞争的市场环境，更好地促进非公有制经济发展。

　　深化资源性产品价格和环保收费改革，是节约能源资源、保护环境、实现可持续发展的重要举措。要扩大用电大户与发电企业直接交易试点，推行居民用电用水阶梯价格制度，健全可再生能源发电定价和费用分摊机制。完善农业用水价格政策。改革污水处理、垃圾处理收费制度。扩大排污权交易试点。在推进这些改革中要注意协调好各方面利益关系，决不能让低收入群众的基本生活受到影响。

　　要继续推进财税体制改革。健全公共财政体系。完善财政转移支付制度，加大一般性转移支付，增强地方政府提供基本公共服务的能力。健全省以下财政管理体制，完善县级基本财力保障机制，推进省直管县财政管理方式改革。全面编制中央和地方政府性基金预算，试编社会保险基金预算，完善国有资本经营预算制度。增强财政预算的透明度。继续做好增值税转型工作。推进资源税改革。统一内外资企业和个人城建税、教育费附加制度。

　　健全金融体系是应对国际金融危机冲击的重要举措。要继续完善国有控股金融机构公司治理，改善经营管理机制，提高风险管控能力。继续推动政策性金融机构改革。稳步推进资产管理公司转型。深化农村信用社改革。推动中小金融机构规范发展。大力发展金融市场，鼓励金融创新。推进跨境贸易人民币结算试点，逐步发展境外人民币金融业务。推进存款保险制度建设。加快发展农业保险。

　　要按照政事分开、事企分开和管办分离的要求，在科学分类的基础上，积极稳妥推进事业单位改革。

　　我们的改革是全面的改革，包括经济体制改革、政治体制改革以及其他各领域的改革。没有政治体制改革，经济体制改革和现代化建设就不可能成功。要发展社会主义民主，切实保障人民当家作主的民主权利，特别是选举权、知情权、参与权、表达权和监督权。进一步扩大基层民主，健全基层自治组织和民主管理制度，让广大群众更好地参与管理基层公共事务。要依法治国，健全法制，特别要重视那些规范和监督权力运行的法律制度建设。创新政府立法工作的方法和机制，扩大立法工作的公众参与。

　　我们要全面适应国际形势变化和国内发展要求，拓展对外开放的广度和深度。

　　稳定发展对外贸易。今年的主要着力点是拓市场、调结构、促平衡。坚持实施市场多元化战略和以质取胜战略，落实和完善出口退税、出口信贷、出口信用保险等各项政策措施，继续改善海关、质检、外汇等方面的服务。巩固传统市场，大力开拓新兴市场。优化出口产品结构，稳定劳动密集型产品出口，扩大机电产品和高新技术产品出口，大力发展服务贸易和服务外包，努力培育出口品牌和营销网络，继续严格控制“两高一资”产品出口。积极推进加工贸易转型升级。促进进出口平衡发展，重点扩大先进技术装备、关键零部件和国内紧缺物资进口，稳定各项进口促进政策和便利化措施，敦促发达国家放宽高新技术产品出口限制。

　　推动利用外资和对外投资协调发展。优化利用外资结构，鼓励外资投向高端制造业、高新技术产业、现代服务业、新能源和节能环保产业，鼓励跨国公司在华设立地区总部等各类功能性机构，鼓励中外企业加强研发合作。鼓励外资参与国内企业改组改造和兼并重组，加快建立外资并购安全审查制度。促进“引资”与“引智”相结合。引导外资向中西部地区转移和增加投资。加快实施“走出去”战略，鼓励符合国外市场需求的行业有序向境外转移产能，支持有条件的企业开展海外并购，深化境外资源互利合作，提高对外承包工程和劳务合作的质量。进一步简化各类审批手续，落实企业境外投资自主权。“走出去”的企业要依法经营，规避风险，防止恶性竞争，维护国家整体利益和良好形象。

　　深化多边双边经贸合作。加强和改善与发达国家的经贸关系，深化与发展中国家的互利合作，认真落实中非务实合作八项新举措。发挥经济高层对话和双边经贸联委会作用。加快自由贸易区建设步伐。积极参与多哈回合谈判，推动早日达成更加合理、平衡的谈判结果。反对各种形式的保护主义，妥善处理贸易摩擦。

　　上海世博会即将拉开帷幕。要加强与各参展国家、地区和国际组织的合作，把上海世博会办成一届成功、精彩、难忘的盛会，办成一届促进人类文明进步，促进科技创新，促进我国与世界各国交流合作、共同发展的盛会。

　　（八）努力建设人民满意的服务型政府

　　一年来，政府自身改革和建设取得新进展。为应对各种困难，我们特别注意发扬民主、倾听基层群众意见，重视维护群众利益。广大公务员兢兢业业、勤勉尽责，为保增长、保民生、保稳定作出了积极贡献。但是政府工作与人民的期望还有较大差距。职能转变不到位，对微观经济干预过多，社会管理和公共服务比较薄弱；一些工作人员依法行政意识不强；一些领导干部脱离群众、脱离实际，形式主义、官僚主义严重；一些领域腐败现象易发多发。我们要以转变职能为核心，深化行政管理体制改革，大力推进服务型政府建设，努力为各类市场主体创造公平的发展环境，为人民群众提供良好的公共服务，维护社会公平正义。

　　我们要全面正确履行政府职能，更加重视公共服务和社会管理。加快健全覆盖全民的公共服务体系，全面增强基本公共服务能力。健全重大自然灾害、突发公共安全事件应急处理机制。加强防灾减灾能力建设。加强食品药品质量监管，做好安全生产工作，遏制重特大事故发生。

　　要适应新形势，推进社会管理体制改革和创新，合理调节社会利益关系。认真解决企业改制、征地拆迁、环境保护、劳动争议、涉法涉诉等领域损害群众利益的突出问题，保障人民群众的合法权益。加强和改进信访工作。改善流动人口管理和服务。加强社会治安综合治理，着力解决突出治安问题，防范和依法严厉打击各类违法犯罪活动，维护国家安全和社会稳定。

　　要努力提高执行力和公信力。坚持决策的科学化、民主化，使各项政策更加符合实际、经得起检验。加强对政策执行情况的检查监督，做到令行禁止。强化行政问责，对失职渎职、不作为和乱作为的，要严肃追究责任。各地区、各部门对中央的决策部署要执行有力，绝不允许各自为政。各级行政机关及其公务员要自觉遵守宪法和法律，严格依法行政。切实改进行政执法工作，努力做到规范执法、公正执法、文明执法。加快建立健全决策、执行、监督相互制约又相互协调的行政运行机制。

　　要把反腐倡廉建设摆在重要位置，这直接关系政权的巩固。各级领导干部特别是高级干部要坚决执行中央关于报告个人经济和财产，包括收入、住房、投资，以及配偶子女从业等重大事项的规定，并自觉接受纪检部门的监督。要把查处违法违纪大案要案，作为反腐败的重要任务。充分发挥监察、审计部门的作用，加强对行政权力运行的监督。要建立健全惩治和预防腐败体系的各项制度，特别要健全公共资源配置、公共资产交易、公共产品生产等领域的管理制度，增强制度约束力。要坚持勤俭行政，反对铺张浪费，不断降低行政成本。严格控制楼堂馆所建设，禁止高档装修办公楼，加快公务接待、公车使用等制度改革，从严控制公费出国出境。切实精简会议和文件，特别要减少那些形式重于内容的会议、庆典和论坛。要深入推进政务公开，完善各类公开办事制度和行政复议制度，创造条件让人民批评政府、监督政府，同时充分发挥新闻舆论的监督作用，让权力在阳光下运行。

　　我们所做的一切都是要让人民生活得更加幸福、更有尊严，让社会更加公正、更加和谐。

　　各位代表！

　　促进民族团结，实现共同进步，是中华民族的生命、力量和希望所在。要巩固和发展平等、团结、互助、和谐的社会主义民族关系。认真落实中央支持少数民族和民族地区发展的政策措施，优先支持边疆民族地区加快发展。加快完成边境一线地区危旧房改造，实施游牧民定居工程。新型农村社会养老保险要优先在边境县、民族地区贫困县试点。加大扶持人口较少民族发展力度。继续推进兴边富民行动。重视保护少数民族文化遗产和民族地区生态环境。切实做好少数民族流动人口公共服务、就业和管理工作，保障他们的合法权益。同时，加强国家意识、公民意识教育，旗帜鲜明地反对民族分裂，维护祖国统一。要让少数民族和民族地区各族群众充分感受到祖国大家庭的温暖。

　　我们要全面贯彻党的宗教工作基本方针，依法管理宗教事务。发挥宗教界人士和信教群众在促进经济发展和社会和谐中的积极作用。

　　我们要认真贯彻党的侨务政策。维护海外侨胞、归侨侨眷的合法权益，支持他们传承中华文化，参与祖国现代化建设和促进和平统一大业。

　　各位代表！

　　过去一年，国防和军队现代化建设取得新的成就。人民解放军和武警部队圆满完成国庆首都阅兵、重点地区维稳等重大任务，为维护国家安全和发展利益发挥了重要作用。新的一年，要紧紧围绕党和国家工作大局，着眼全面履行新世纪新阶段军队历史使命，按照革命化现代化正规化相统一的原则，加强军队全面建设。以增强打赢信息化条件下局部战争能力为核心，提高应对多种安全威胁、完成多样化军事任务的能力。大力加强军队思想政治建设。加快全面建设现代后勤步伐。加强国防科研和武器装备建设。依法治军、从严治军，提高军队正规化水平。积极稳妥地深化国防和军队改革。加强武警部队现代化建设，增强执勤、处置突发事件、反恐、维稳能力。加强国防动员和后备力量建设。各级政府要一如既往地关心支持国防和军队建设，巩固和发展军政军民团结。

　　各位代表！

　　我们将坚定不移地贯彻“一国两制”、“港人治港”、“澳人治澳”、高度自治的方针，全力支持香港、澳门保持长期繁荣稳定。支持香港巩固并提升国际金融、贸易、航运中心地位，发展优势产业，培育新的经济增长点。支持澳门发展旅游休闲产业，促进经济适度多元化。要认真实施珠江三角洲地区改革发展规划纲要，积极推进港珠澳大桥等大型跨境基础设施建设和珠海横琴岛开发，深化粤港澳合作，密切内地与港澳的经济联系。伟大祖国永远是香港、澳门的坚强后盾。只要特别行政区政府与各界人士同心协力，包容共济，共同维护繁荣稳定发展的大局，香港、澳门的明天一定会更加美好。

　　过去的一年，两岸关系在新的历史起点上取得重要进展，呈现和平发展良好势头。两岸交流合作不断深入，全面直接双向“三通”得以实现。经济关系正常化迈出重要步伐，经济合作制度化建设逐步推进。两岸关系持续改善和发展，给两岸同胞带来了实实在在的利益。在新的一年里，我们要继续坚持发展两岸关系、促进祖国和平统一的大政方针，牢牢把握两岸关系和平发展的主题，不断开创两岸关系和平发展新局面。密切两岸经贸金融交往，深化产业合作，支持在大陆的台资企业发展，维护台胞合法权益。鼓励有条件的大陆企业赴台投资。支持海峡西岸经济区在两岸交流合作中发挥先行先试作用。通过商签两岸经济合作框架协议，促进互利共赢，建立具有两岸特色的经济合作机制。拓展文化教育交流，共同弘扬中华文化。加强两岸民众和社会各界交流，共同分享两岸关系和平发展成果，进一步凝聚推动两岸关系和平发展的共识。坚持大陆和台湾同属一个中国，巩固两岸关系和平发展的政治基础，增强两岸政治互信。我们坚信，全体中华儿女同心协力，祖国完全统一的宏伟大业一定能够实现！

　　各位代表！

　　过去的一年，外交工作取得新的重大成绩。我国积极参加应对国际金融危机、气候变化等国际合作，在一系列重大多边会议上发挥了独特的建设性作用。我们积极开展全方位外交，与各大国、周边国家和广大发展中国家的对话合作稳步推进。大力加强人文等领域外交。有效维护我国公民和法人在海外的合法权益。

　　新中国外交走过了60年光辉历程，在国际舞台上留下了闪光足迹。我们将继承发扬光荣传统，始终高举和平、发展、合作旗帜，坚持独立自主的和平外交政策，坚持走和平发展道路，奉行互利共赢的开放战略，推动建设持久和平、共同繁荣的和谐世界，为我国现代化建设创造良好的外部环境。新的一年，我们将继续以20国集团金融峰会等重大多边活动为主要平台，积极参与国际体系变革进程，维护发展中国家利益。统筹协调好双边外交与多边外交、国别区域外交与各领域外交工作，推动我国与各大国、周边国家和发展中国家的关系全面深入发展。紧紧抓住中国－东盟自贸区全面建成、上海合作组织召开峰会等契机，积极推进区域合作。进一步做好应对气候变化、能源资源合作等方面的对外工作，在妥善解决热点问题和全球性问题中发挥建设性作用。中国政府和人民愿与国际社会携手努力，共同应对风险挑战，共同分享发展机遇，为世界和平与发展作出新贡献！

　　各位代表！

　　艰辛成就伟业，奋斗创造辉煌。让我们在以胡锦涛同志为总书记的党中央领导下，凝聚起亿万人民的智慧和力量，再接再厉，开拓进取，全面完成“十一五”规划的各项任务，不断夺取改革开放和社会主义现代化建设事业的新胜利！
